# Effects of the Share 35 Rule on Waitlist and Liver Transplantation Outcomes for Patients with Hepatocellular Carcinoma

**DOI:** 10.1371/journal.pone.0170673

**Published:** 2017-01-25

**Authors:** Kristopher P. Croome, David D. Lee, Denise Harnois, C. Burcin Taner

**Affiliations:** Department of Transplant, Mayo Clinic Florida, Jacksonville, Florida; Universidad de Navarra, SPAIN

## Abstract

**Introduction:**

Several studies have investigated the effects following the implementation of the “Share 35” policy; however none have investigated what effect this policy change has had on waitlist and liver transplantation (LT) outcomes for hepatocellular carcinoma(HCC).

**Methods:**

Data were obtained from the UNOS database and a comparison of the 2 years post-Share 35 with data from the 2 years pre-Share 35 was performed.

**Results:**

In the pre-Share35 era, 23% of LT were performed for HCC exceptions compared to 22% of LT in the post-Share35 era (p = 0.21). No difference in wait-time for HCC patients was seen in any of the UNOS regions between the 2 eras. Competing risk analysis demonstrated that HCC candidates in post-Share 35 era were more likely to die or be delisted for “too sick” while waiting (7.2% vs. 5.3%; p = 0.005) within 15 months. A higher proportion of ECD (p<0.001) and DCD (p<0.001) livers were used for patients transplanted for HCC, while lower DRI organs were used for those patients transplanted with a MELD≥35 between the 2 eras (p = 0.007).

**Conclusion:**

No significant change to wait-time for patients listed for HCC was seen following implementation of “Share 35”. Transplant program behavior has changed resulting use of higher proportion of ECD and DCD liver grafts for patients with HCC. A higher rate of wait list mortality was observed in patients with HCC in the post-Share 35 era.

## Introduction

The Model for End Stage Liver Disease (MELD) has been used as the method of liver graft allocation since 2002. Liver grafts are distributed geographically based on MELD score in Organ Procurement and Transplant Network (OPTN) sharing areas consisting of local, regional and national tiers. It has been shown that patients with a MELD score ≥ 35 have wait list mortality rates that were similar to Status 1 candidates, who represent a cohort of candidates with acute liver failure most likely to die within 7 days without liver transplant [1]. To facilitate liver graft allocation to those patients with high wait list mortality, regional sharing for patients with a MELD ≥ 35 was implemented in June of 2013 with the goal of increasing life saving liver transplant for the sickest patients and decreasing death on the waiting list (“Share 35”).

Initial publications looking at the overall effects of “Share 35” demonstrated no reduction in wait list mortality in the 2 years following its implementation [2,3]. When a subanalysis was performed looking at patients with MELD ≥ 35, a reduction of 90 day waitlist mortality (66% versus 59%) was observed. While this reduction was heralded as an encouraging result, some authors have stressed that the effects of any policy change must be examined for all patients awaiting LT [3].

LT has been established as an effective treatment for patients with hepatocellular carcinoma (HCC) within specific size criteria (Milan criteria) since the seminal report by Mazzaferro and colleagues [4]. Patients with HCC within Milan criteria receive an “exception” MELD score, in an attempt reflect candidates expected wait list mortality due to tumor progression. Multiple previous studies have suggested that non-HCC candidates have significantly higher wait list drop-off rates than HCC patients due to mortality and clinical deterioration [5–7]. The primary goal of the present study was to assess the impact of the Share 35 policy on patients undergoing LT for HCC at the 2 years mark post-implementation.

## Materials and Methods

After approval from the Mayo Clinic Institutional Review Board, data were obtained and extracted from the United Network of Organ Sharing (UNOS) Standard Analysis and Research file. The study population included all patients on the waitlist for LT in the United States from June 18, 2011 to June 18, 2015. Prior to Share 35 in the United States liver allografts were allocated to patients with the highest MELD score in sequential sharing areas consist of first local, then regional, and finally national tiers. The Share 35 policy was implemented in June 2013 to achieve broader sharing whereby the sickest waitlist candidates (patients with a MELD score ≥ 35) are first prioritized in a tiered manner regionally before any local candidates with MELD scores less than 35 are offered the livers. For the majority of analyses, data were provided for 2 eras; the 2 years pre-implementation of “Share 35” (Era 1) (6/18/2011 to 6/17/2013) and the 2 years post-implementation (Era 2) (6/18/2013 to 6/18/2015). Share 35 was implemented in June 2013 and therefore the dates were chosen so that we had 2 equal time periods with a minimum of 1 year of follow-up.

Donor and recipient factors were examined, including all the components of the liver donor risk index (DRI), donor sex, donor body mass index (BMI), recipient age, recipient BMI, recipient sex, recipient etiology of liver disease, biologic Model for End-Stage Liver Disease (MELD) score at transplant, match MELD score at transplant (match MELD is the score with which the organ was allocated; it also includes exception scores for cancers or other indications), presence of hepatocellular cancer (HCC) as a secondary diagnosis, re-transplantation, mechanical ventilation at the time of transplant and medical condition at the time of transplant [8]. Extended criteria grafts (ECD) were defined as a DRI > 1.7 [9,10].

Graft survival was calculated from the time of transplant until death, graft loss, or date of last follow-up. The occurrence and the date of death were obtained from data reported to the Scientific Registry of Transplant Recipients (SRTR) by transplant centers and were completed by data from the US Social Security Administration and from the Organ Procurement Transplant Network (OPTN).

Wait-list outcomes were analyzed with previously defined methods [2,11]. Briefly, removals for death as well as for “too sick” were treated as deaths. Patients that had tumor progression were included in the delisting for “too sick” definition. Patients’ wait list status was therefore classified into 1 of 3 categories: death, transplanted or still waiting/other. For the wait-list analysis a wash-out period was used. The pre-Share 35 era cohort listing dates were shortened to avoid overlap with the post-Share 35 cohort by 180 days. The Post-Share 35 cohort was also shortened in a similar manner, so that both eras were equal time intervals.

All statistical analyses were performed using STATA 12 (Stata Corp., College Station, TX). Differences between groups were analyzed using the unpaired *t* test for continuous variables and by the χ^2^ test or continuity correction method for categorical variables. Wilcoxon rank-sum was used for variables that did not display a normal distribution. Survival curves for patient or graft survival were generated using the Kaplan-Meier method and compared by the log-rank test. All statistical tests were two-sided and differences were considered significant when p < 0.05.

## Results

In Era 1 a total of 12,636 LT were performed of which 2916 (23.0%) were performed for HCC compared to 13533 LT of which 3029 (22.4%) were for HCC in Era 2. There was no difference in the proportion of LTs performed for HCC in the 2 eras (p = 0.18). No difference in the median match MELD score was seen for HCC patients between Era 1 and Era 2 (25 [range 6–40] vs. 25 [range 6–40]; p = 0.12). For HCC patients the median wait-time in era 1 was 185 days compared to 195 days in era 2. No significant difference in wait list time for HCC patients was seen between Era 1 and Era 2 in any of the eleven UNOS regions. Similarly, no difference in wait list time was seen for non-HCC patients in any of the eleven UNOS regions except for Region 1 where wait time increased (206 vs. 277 days; p = 0.02). When a sub-analysis was performed for all patients with a MELD ≥ 35, no difference in wait list time was seen for any of the eleven Regions. There was a slight statistical increase in the proportion of patients newly listed with HCC exception MELD between the 2 eras (14.3% vs.15.0%; p = 0.04); however this statistical difference is of minimal clinical relevance.

Data was analyzed to determine the number of cases in each Region in which organs were regionally shared for HCC exception MELD scores ≥ 35 (“Share 35”). In Era 1, prior to “share 35” policy implementation, there were no organs shared for this reason. In Era 2, no organs were regionally shared for HCC exception MELDs ≥ 35 in Regions 3, 4, 6, 7, 10, 11. In Regions 1, 2, 8, and 9 less than 2% or less of liver grafts were regionally shared for HCC exception MELD scores ≥ 35 (Region 1: n = 3 [2%]; Region 2: n = 2 [0.6%]; Region 8: n = 2 [0.9%]; Region 9: n = 2 [1.2%]). In Region 5, 33 [8.3%] liver grafts were regionally shared for exception MELD scores ≥ 35.

**Fig 1:**
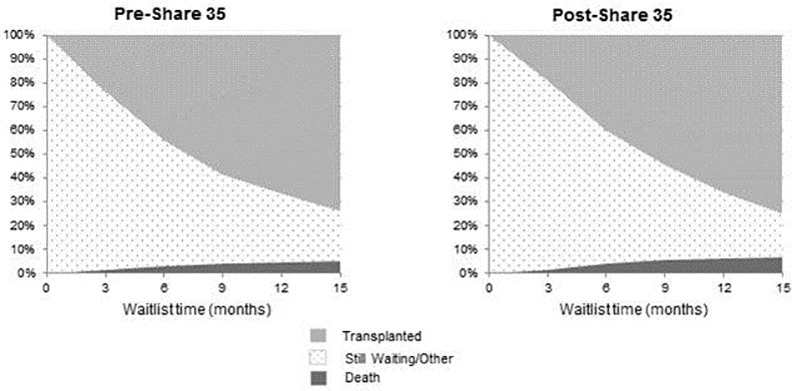
Wait list outcomes in patients undergoing liver transplantation for hepatocellular carcinoma in Era 1 (Pre-Share 35) and Era 2 (Post-Share 35). Candidates in Era 2 were more likely to die while waiting (7.2% vs. 5.3%; p = 0.005) within 15 months, while there was no difference in the overall likelihood of getting transplanted (75% vs. 74%; p = 0.42).

**Fig 2:**
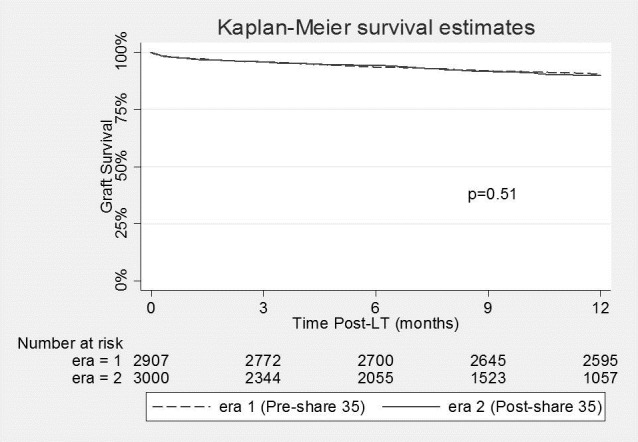
Graft survival in patients undergoing liver transplantation for hepatocellular carcinoma in Era 1 (Pre-Share 35) and Era 2 (Post-Share 35).

**Fig 3:**
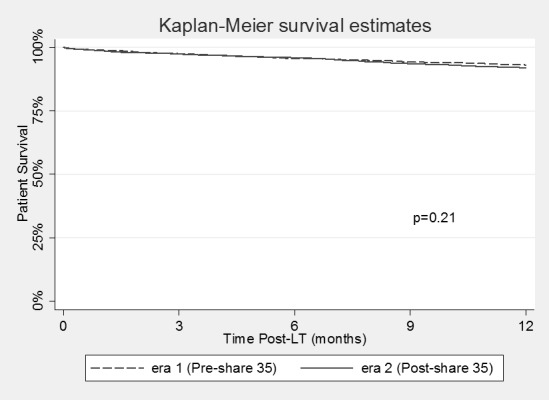
Patient survival in patients undergoing liver transplantation for hepatocellular carcinoma in Era 1 (Pre-Share 35) and Era 2 (Post-Share 35).

Competing risk analysis for patients’ wait-listed for HCC in the two eras can be seen in **Fig 1**. Candidates in Era 2 were more likely to die while waiting (7.2% vs. 5.3%; p = 0.005) within 15 months, while there was no difference in the overall likelihood of getting transplanted (75% vs. 74%; p = 0.42). Change in the death rate for waitlisted HCC patients varied significantly between regions. Regions 4 and 5 had the largest increase (5% to 11%; p = 0.006 and 7% to 14%; p = 0.001, respectively) while Region 3 saw a reduction in waitlist mortality (5% to 2%; p = 0.06). Post-transplant graft survival by 12 months for patients with HCC did not change between the 2 eras (p = 0.51), nor did post-transplant patient survival (p = 0.21) (**Figs 2 and 3**).

Patients with a MELD score ≥ 35 were less likely to die while waiting in era 2 (28%) compared to era 1 (31%) (p<0.001) and were more likely to have been transplanted (42% vs 30%; p<0.001). For non-HCC patients with a MELD <35 there was no difference in mortality rate between era 1 and era 2 (10% vs 11%; P = 0.11), while there was a lower likelihood of getting transplanted (52% vs. 46%; p<0.001).

**Fig 4:**
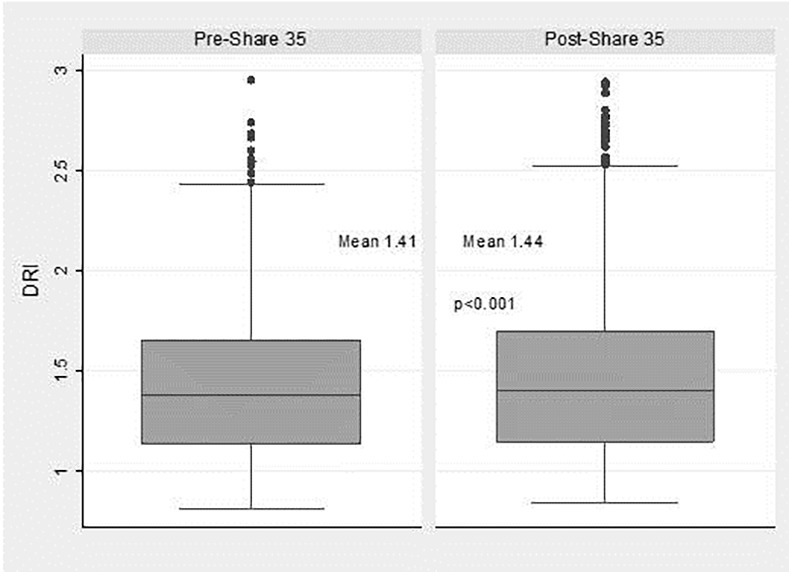
Distribution of donor risk index in patients undergoing liver transplantation for hepatocellular carcinoma in Era 1 (Pre-Share 35) and Era 2 (Post-Share 35).

Mean donor DRI for patients transplanted for HCC increased from 1.41 to 1.44 (p < 0.001), while DRI decreased for those patients transplanted with a MELD ≥ 35 between Era 1 and Era 2 (1.44 vs. 1.40; p = 0.007). Patients transplanted for HCC received a higher proportion of ECD grafts (21.1% vs. 25.0%; p < 0.001), grafts from DCD donors (5.9% vs. 8.5%; p < 0.001) and grafts from PHS increased risk donors (13.3% vs. 21.8%;p < 0.001) between Era 1 and Era 2 (**Table 1**). Recipient characteristics in patients undergoing LT for HCC in Era 1 and Era 2 can be seen in **Table 2**. Patients transplanted with a MELD ≥ 35 had a decreased proportion of ECD organs (20.2% to 16.8%) between Era 1 and Era 2. Non-HCC patients with a MELD<35 had a slight non-significant trend of increased proportion of ECD grafts (22.6% vs 24.0%; p = 0.06) between the Eras. The distribution of graft DRI for HCC patients undergoing LT in the 2 eras can be seen in **Fig 4**.

**Table 1:** Donor characteristics in patients undergoing liver transplantation for hepatocellular carcinoma in Era 1 (Pre-Share 35) and Era 2 (Post-Share 35).

	Pre-Share 35	Post-Share 35	p-value
	N = 2916	N = 3029	
DRI	1.41 ± 0.34	1.44 ± 0.36	<0.001
ECD (DRI > 1.7)	615 (21.1%)	757 (25.0%)	<0.001
Donor age ≥ 70 years	111 (3.8%)	145 (4.8%)	0.06
DCD	172 (5.9%)	257 (8.5%)	<0.001
PHS Increased Risk	388 (13.3%)	660 (21.8%)	<0.001
Share Type			
Local	2420 (83%)	2393 (79%)	<0.001
Regional	437 (15%)	545 (18%)	0.002
National	58 (2%)	121 (4%)	<0.001
Regional Share 35	NA	42 (1.4%)	NA

DRI: Donor Risk Index; ECD: Extended Criteria Donor; DCD: Donation after Cardiac Death Donor; PHS: Public Health Service.

**Table 2:** Recipient characteristics in patients undergoing liver transplantation for hepatocellular carcinoma in Era 1 (Pre-Share 35) and Era 2 (Post-Share 35).

	Pre-Share 35	Post-Share 35	
Recipient Characteristics	N = 2916	N = 3029	p value
**Age at transplant (years)**	58.8 ± 7.6	60.0± 7.3	<0.001
**Body mass index**	28.6± 5.3	28.6± 5.2	>0.99
**Gender (male)**	2227 (76%)	2333 (77%)	0.55
**Diagnosis**			
Hepatitis C virus serology	1875 (64%)	1877 (62%)	0.06
EtOH	52 (1.6%)	56 (1.9%)	0.85
NASH	46 (1.6%)	67 (2.2%)	0.07
**Calculated MELD score†**	12 (6–48)	11 (6–50)	0.09††
**Match MELD score†**	25 (6–40)	25 (6–40)	0.12††
**Race/ethnicity**			
White	1977 (68%%)	2063 (68%%)	0.80
Black	282 (9.7%)	317 (10%)	0.31
other	657 (23%)	649 (21%)	0.30

†median (range)

††Wilcoxon rank-sum used.

## Discussion

Broader regional sharing though the “Share 35” policy change was implemented with the goal of increasing life saving LT for the sickest patients on the wait list. Initial publications examining the effects of “Share 35” have shown several positive results, including reduction in 90-day mortality for patients with MELD scores ≥ 35 [2,12]. While these initial results are encouraging, it is important to fully explore the effects of “Share 35” for all patient groups.

The present study specifically examined the effects of “Share 35” on patients who underwent LT for HCC by comparing the 2 years pre-implementation of “Share 35” with the 2 years post-implementation. The proportion of LT performed for patients with HCC did not change following implementation of “Share 35” nor did the waiting time. Despite these findings, a higher rate of death/delisting for “too sick” for patients on the wait list for HCC in the post “Share 35” era (5.3% vs. 7.2% at 15 months) was observed. Given the relatively stable number of available liver grafts, this finding highlights the reality that higher transplant rates for one cohort of patients inevitably results in less organ availability for another. The present study did demonstrate a slight increase in the proportion of patients newly listed with HCC MELD exception score between the 2 periods (14.3% vs.15.0%). This increase in listed HCC patients, associated with no increase in the proportion of LT performed for patients with HCC, likely accounts for the higher death rate for HCC patients on the wait list. It must, however, be acknowledged that this rate of death is still significantly lower than the death rate for patients with high biologic MELD scores and perhaps is an unavoidable consequence of attempts to reduce the disparity in waitlist mortality for HCC and non-HCC patients awaiting LT [13]. It will also be important to follow results of wait listed patients with HCC in light of the recently implementation changes to HCC MELD exception points nationally. Modelling that was performed prior to the policy change that resulted in a 6 month delay in receiving HCC exception points, was designed to increase dropout (death) for patients with HCC on the waiting list. These models demonstrated an increase in wait list death for patients with HCC and a decrease in wait list death for non-HCC patients [14]. Proponents of the new HCC exception point policies would argue that HCC patients who died/dropped off the wait list likely had more advanced tumor biology and would have had an inferior result if they had been transplanted [15]. While this may be the case with some patients it should also be noted that modelling demonstrated that with longer delays, the biological MELD for HCC patients at transplant also increased [14]. It is conceivable that some patients with HCC that died while waiting may also have died as a result of their underlying liver disease.

The newly implemented modifications to the MELD exception points to patients with HCC also created a cap to the exception score of 34, to avoid the allocation of regionally “Share 35” organs to patients with exception MELD scores ≥ 35. The present study demonstrated that almost no patients received regionally shared liver grafts for MELD exception scores ≥ 35 in any of the eleven UNOS regions except Region 5. In Region 5, 8.3% of LT for HCC received liver grafts as a result of “Share 35”. With capping of MELD exception at 34 for patients with HCC, for regions with high allocation MELD scores, it will become even more imperative to attempt to increase the usage of higher DRI organs such as DCD grafts for patients with HCC.

Another important finding of the present study was that between the 2 eras studied, there was a change in the liver graft quality that HCC patients received. An increase the usage of ECD and DCD livers was observed for patients with HCC undergoing LT while a decrease was observed for patients with a MELD score ≥ 35. This finding suggests a perhaps expected shift of the higher quality liver grafts allocated to patients with the highest MELD scores. In order to maintain stable wait times for patients with HCC, in the post-implementation of “Share 35” era, transplant programs have adjusted their behavior by using more ECD and DCD organs for these patients. Increased death rates for patients on the wait list for HCC may also have played a role in the acceptance of more ECD and DCD grafts for this cohort. A previous study examining the effects of “Share 35” at 1 year post implementation demonstrated liver grafts with higher DRI were more likely to be shared within a region in pre-Share 35 era, while in post-Share35 era, liver grafts with lower DRI were more likely to be shared [12].

Previous studies examining the effects of “Share 35” have demonstrated no overall change in the DRI or CIT of liver grafts used for LT [2]. We also did not find any overall increase in DRI when all transplants were examined. This suggests “Share 35” did not increase the total number of higher DRI organs being used, but instead simply resulted in a modification in which recipients these organs are being used for. It should be noted, that despite no substantial change in DRI, initial publications on “Share 35” did show a small decrease in overall liver graft discard rates [2]. This may suggest that by optimizing donor-recipient selection we can perhaps more effectively utilize higher DRI liver grafts while still maintaining good post-transplant outcomes. Patients with HCC who have relatively preserved synthetic function may represent ideal candidates for DCD or other higher DRI organs (ECD and DCD) as they can more safely “weather the storm” of mild delayed graft function [9]. Indeed, previous authors have demonstrated excellent results for patients with HCC receiving DCD grafts [16]. Despite the use of a higher proportion of ECD and DCD organs for patients with HCC, the present study did not show any change in graft or patient survival by 12 months for these patients when comparing the 2 eras. This may suggest that as we continue to search for new ways to increase the available liver donor pool, patients with HCC may represent a suitable group to use higher DRI organs. The increased wait list death rate for this population may also change dogmatic thinking that HCC patients have time to wait for a better organ.

Limitations of the present study include its reliance on registry data and lack of granularity that can be obtained at single program level. In addition, due to the fact that data was only available for 2 years following implementation of “Share 35”, many outcomes were truncated at shorter follow-up intervals.

In conclusion, the present study demonstrates that there has been no significant change to wait time for patients listed for HCC following implementation of “Share 35”. Transplant program behavior has changed resulting use of a higher proportion of ECD and DCD liver grafts for patients with HCC, while lower DRI liver grafts are preferentially allocated to patients with higher MELD scores through regional “Share 35”. Despite these changes, no significant difference in post-transplant graft or patient survival has been observed. A higher rate of waitlist mortality was observed in patients with HCC in the post-“Share 35” era, however, wait list mortality rate still remains substantially lower than that observed for non-HCC patients.

## Abbreviations

BMI: Body mass index
CIT: Cold ischemia time
DCD: Donation after Cardiac Death
DBD: Donation after Brain Death
ECD: Extended Criteria Donor
HCC: Hepatocellular carcinoma
LT: Liver transplant
MELD: Model for end stage liver disease
SD: Standard deviation
